# Hemiparesis after Operation of Astrocytoma Grade II in Adults: Effects of Acupuncture on Sensory-Motor Behavior and Quality of Life

**DOI:** 10.1155/2013/859763

**Published:** 2013-06-24

**Authors:** Haibo Yu, Sven Schröder, Yongfeng Liu, Zhifeng Li, Ying Yang, Yu Chen, Xingxian Huang

**Affiliations:** ^1^Shenzhen Traditional Chinese Medicine Hospital, Shenzhen City, Guangdong 518033, China; ^2^HanseMerkur Zentrum für Traditionelle Chinesische Medizin am Universitätsklinikum Hamburg-Eppendorf, 20246 Hamburg, Germany

## Abstract

To evaluate the effect of acupuncture on hemiparesis and quality of life for adults with brain astrocytoma grade II, we conducted a randomized, observer-blinded clinical trial. Fifty-eight patients were randomized to *standard rehabilitation (SR) therapy without acupuncture* (*n* = 20), *SR plus standard acupuncture (SA)* (*n* = 19), and *SR plus individualized acupuncture (IA)* (*n* = 19). SA points were *PC6*, *SP6*, *HT1*, *LU5*, *BL40*, and *ST36*, while a special concept called “*connecting and regulation Ren and Du*” and “*Jin-3-needling*” served as IA. This treatment was individualized according to the clinical syndrome. The outcome was measured by the Barthel Index (BI), the Fugl-Meyer scale (FM), and the EORTC Core Quality of Life Questionnaire (QLQ-C30) with the Brain Cancer Module (BCM20). IA + SR reached significantly higher BI scores than SA + SR, which reached significantly higher BI scores than SR. IA + SR was significantly superior to SA + SR and to SR at the 8th week for the scores of FM motor and sensory assessments and most QLQ-C30-BCM20 items. In conclusion, the individualized acupuncture concept of “*connecting and regulating Ren and Du*” combined with “*Jin-3-needling*” offers a promising possibility for the treatment of hemiparesis due to astrocytoma, but further evaluation is mandatory.

## 1. Introduction

Astrocytic tumors comprise a wide range of neoplasms of the central nervous system (CNS) which shows invasive and progressive growth. The following clinicopathological entities can be distinguished as pilocytic astrocytomas (grade I), fibrillary astrocytomas (grade II), anaplastic astrocytomas (grade III), and glioblastoma multiforme (grade IV) [1]. The most common site of these tumors is the brain [2]. They make up about 42% of all brain tumors including benign tumors and 80% of all malignant brain tumors [3]. These tumors tend to grow and infiltrate into the normal brain tissue, which makes complete surgical removal very difficult, or usually impossible, and complicates treatment [4]. The World Health Organization (WHO) classifies astrocytomas into the previously described four grades depending on how fast they are growing and the likelihood that they will spread (infiltrate) to nearby brain tissue [5]. Grade II astrocytomas are also called low-grade astrocytomas or diffuse astrocytomas and are usually infiltrating tumors. These tumors grow relatively slow and usually do not have well-defined borders. They are generally more common in men and are most common in the cerebral hemispheres of young adult patients [6]. 

Patients with brain tumors have many problems which relate to the consequences of neurological dysfunction caused by a destructive or invasive tumor mass in the central nervous system. Astrocytic tumors can cause several neurological symptoms such as sensorimotor deficit, ataxia, language problems, and cognitive decline [7]. Symptoms can arise from the disease process itself, which usually causes focal neurological damage, and the side effects of treatment (surgery, radiation therapy, and chemotherapy) can cause more diffuse damage [6]. Nevertheless, it must be emphasized that although more than 60 percent of patients with astrocytic tumors suffer from hemiparesis at the time of diagnosis, but only 3 percent complain of weakness as the initial symptom. At the onset of their disease, patients with gliomas have relatively low rates of hemiparesis and hemianesthesia, by the time of diagnosis, some or all of these findings are present in the majority of patients [8]. 

For more than 3000 years, practitioners in China have used acupuncture to treat various diseases, including hemiparesis [9]. Acupuncture treatment also has become increasingly popular in western societies. Acupuncture, as a treatment of individuals with hemiplegia after stroke, has a long tradition in China and can improve functions and the quality of life. Recent studies showed benefits in the rehabilitation of strokes [10–12]. But while there are still limitations in the quality [13], further controlled trials have been recommended and recommendations for study designs have been published [14]. 

The reporting of the promising results of acupuncture on hemiparesis after a stroke to other causes of hemiparesis is unclear and has not been investigated on a large scale in patients with astrocytic brain tumors. Even though acupuncture is a common treatment option for brain tumors in China [15, 16], no randomized, controlled studies have yet explored the effectiveness of acupuncture on the treatment of hemiparesis due to astrocytoma type II. 

Therefore, the aim of this study was to verify whether acupuncture can significantly improve sensory-motor behavior and the quality of life for hemiplegia patients with astrocytoma grade II. While new protocols for hemiparesis after a stroke recommend a standard concept for the selection of acupuncture points [14], the effect of the points on the basis of the prominent symptomatology for an individualized therapy might offer additional treatment effects. 

In Chinese medicine theory hemiplegia, which is caused by brain tumors, has a similar pathogenesis to that described after a stroke by the Chinese medicine terms “wind”, “phlegm”, “blood stasis”, “deficiency”, and “*yang*-hyperactivity” as the main pathological factors. Previous studies showed that acupuncture treatment by needling the *Ren* and *Du* meridians can effectively improve motor disturbance for hemiplegia patients affected by a stroke and enhance the quality of life [17, 18]. Another unique therapy concept is called *Jin-3-needling* technique, introduced by Professor Jin Rui of the Guangzhou University of TCM. On the basis of the experiences of modern medicine, Jin Rui has drawn the development of traditional acupuncture prescriptions into a rational and scientific direction [19]. In his concept, groups of three different acupuncture points are combined for certain physical conditions. This concept is widely applied in various kinds of motor dysfunctions such as hemiplegia due to a stroke [9].

Thus, our study attempts to observe the effectiveness of acupuncture on the quality of life and functional gain and to assess whether a special individualized acupuncture concept is superior to a standard acupuncture concept. 

## 2. Methods

### 2.1. Subjects

#### 2.1.1. Design and Setting

The study was a randomized, observer-blinded, controlled clinical trial with three parallel groups carried out in the Acupuncture Department of the Rehabilitation Center in Shenzhen Hospital Affiliated to Guangzhou University of Traditional Chinese Medicine. All study participants signed a written informed consent before enrollment. The protocols were approved by the Institutional Review Board and Ethics Committee at Shenzhen Hospital Affiliated to the Guangzhou University of Traditional Chinese Medicine (approval number SZSZYY2008/AC/04) and examined in accordance with the guidelines on good clinical practice and ethical standards for human experimentation established by the Declaration of Helsinki.

**Figure 1:**
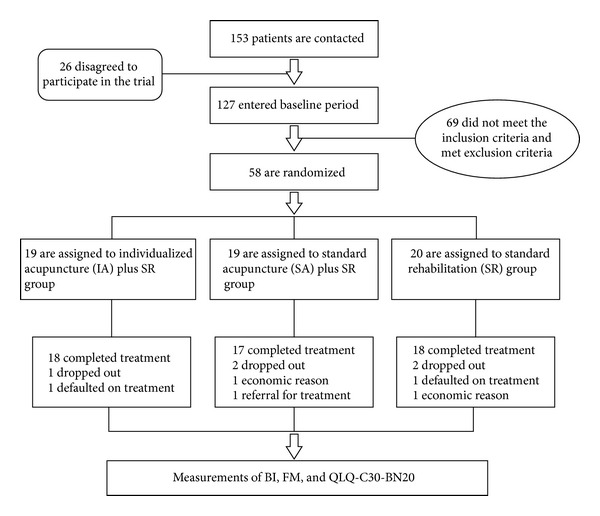
Trial flow diagram.

Patients who had been admitted to the Rehabilitation Center in Shenzhen Hospital Affiliated to the Guangzhou University of Traditional Chinese Medicine after a brain tumor operation of an astrocytoma between September 2008 and December 2012 had been screened (Figure 1).

*Inclusion Criteria*. After the initial screening evaluation, patients were enrolled in the study if they met all of the following criteria: (1) patients affected by hemiparesis with histological evidence of newly diagnosed WHO grade II brain astrocytoma; (2) subjects aged between 18 and 70 years; (3) minimum of 2 weeks after brain tumor surgery, minimum of 4 weeks since the last dose of chemotherapy (6 weeks since nitrosoureas), and at least 6 weeks after radiotherapy before entering the study; (4) Karnofsky performance status (KPS) [20] of 50~90; and a life expectancy of more than 3 months. 

*Exclusion Criteria*. Patients were excluded from the study if the following criteria were true: (1) severe concomitant diseases reducing life expectancy or influencing neurological deficit; (2) pregnancy or breast-feeding women; (3) tumor crossed the midline; and (4) psychiatric disease or mental confusion.

In addition, patients were ineligible if they had a recurrent glioma at the beginning of the study or during the study (CT scan or MRI of the brain confirmed). A complete physical examination and neurological assessment and a thorough inspection on history of hemiplegia were conducted before the trial. The neurosurgeon classified the macroscopic extent of resection at the time of surgery as: biopsy, resection with less than 50% tumor removal, 50–89% tumor removal, or 90–100% tumor removal.

#### 2.1.2. Randomization and Blinding

Centralized random allocation by telephone was performed immediately before the first treatment. Randomization lists were prepared from computer-generated random numbers through DAS (Drug And Statistics) soft 2.0 by staff unconnected with the study and held at a hospital pharmacy. 

The rating scales were evaluated by an investigator who did not have access to the randomization code until all data had been entered. The clinician examined the subjects in a separate room and remained unaware of their group allocation. The consultation with the physician acupuncturist or rehabilitation therapist was standardized in terms of examination, treatment, and permitted discussion. Discussion with the acupuncturist about acupuncture or with the physiotherapist about rehabilitation was not permitted. Statisticians did not participate in the implementation process of the study either. During the preenrollment phase, principal investigators developed a manual of procedures that described the standardized data collection method for all outcome measurement tools, including the Barthel Index, the Fugl-Meyer scale, and the quality of life scale.

#### 2.1.3. Interventions

Patients were randomized before the treatment to either a standard rehabilitation group (SR), a standard acupuncture plus standard rehabilitation group (SA + SR), or an individualized acupuncture plus standard rehabilitation group (IA + SR) by means of a random number table. While the study was conducted at the Acupuncture Department and Rehabilitation Center in Shenzhen Hospital Affiliated to the Guangzhou University of Traditional Chinese Medicine, subjects in the whole study received routine treatment from the Rehabilitation Center in Shenzhen Hospital including activating and passive range of motion (ROM) exercises of the upper and lower extremities for 40 minutes once a day during the full treatment period [21, 22] and received conventional physiotherapy to restore the normal movement and improve the muscle strength [23]. The treatment was customized by a qualified physiotherapist who was blinded to this study according to the individual health/recovery status. Participants received six 60-minute sessions per week of standard physiotherapy. 

Routine medication included antihypertensive, antiepileptic, hypoglycemic, and antihyperlipidemic drugs which were taken according to the advice of a physician depending on different symptoms of each patient.

All subjects in our study were inpatients who had been hospitalized for 2 months. They received 2 months of treatment at the rehabilitation ward, so every patient received a weekly examination or evaluation in our hospital. Patients of the acupuncture groups were treated with acupuncture once a day, 6 times per week for 8 weeks. 

Two appropriately qualified and experienced practitioners were involved in the study. They were physician acupuncturists of the Acupuncture Department in Shenzhen Hospital Affiliated to Guangzhou University of Traditional Chinese Medicine with 10 years of experience after graduation from the University of Traditional Chinese Medicine. 

### 2.2. Acupuncture Treatment

#### 2.2.1. Standard Acupuncture Treatment

Acupuncture therapy in the SA + SR group was given at acupoint *Neiguan (PC6)*, *Sanyinjiao (SP6)*, *Jiquan (HT1)*, *Chize (LU5)*, *Weizhong (BL40)*, and *Zusanli (ST36)*. This acupuncture protocol was developed according to traditional Chinese medicine theory and has been proved to be effective in treating patients with disabled states subsequent to a cerebrovascular lesion [24–26]. Each point was performed on the affected limbs with the even reinforcing-reducing method [27]. The needle was maintained for 30 minutes. All acupuncture treatments were administered by the same physician acupuncturist in this group. 

#### 2.2.2. Individualized Acupuncture Treatment

Patients in IA + SR group were treated according to two specific treatment strategies called “*connecting and regulating Ren Du*” [28] and “*Jin-3-needling*” technique [9] as well as additional points selected on the basis of syndrome differentiation [29].

1. Following the concept of “connecting and regulating *Ren and Du meridian*”: *Guanyuan (RN 4)*, *Qihai (RN 6)*, *Zhongwan (RN 12)*, *Baihui (DU 20)*, and *Renzhong (DU 26) *were selected and acupuncture reinforcement manipulation [27] was applied to the acupoints of *Guanyuan (RN 4)*, *Qihai (RN 6)*, *Zhongwan (RN 12)*, and* Baihui (DU 20). *The acupuncture reduction manipulation [27] was used at* the Renzhong (DU 26) *point.
2. These were combined with points following the concept of “*Jin-3-needling*” [19].
3. Points of *Jin-3-needling* received ipsilateral acupuncture according to the affected limbs as described in Table 1. If there was spasticity, acupuncture reduction manipulation was used at the selected points. If there was no relevant increased muscle tone acupuncture reinforcement manipulation was used at the selected points.
4. Additionally, points chosen on the basis of Chinese syndrome differentiation were added [29]. These syndromes and the additional points are described in Table 2. Points selected for deficiency syndromes (*Yin*-deficiency, *Qi*-deficiency) were stimulated with the acupuncture reinforcement manipulation and excess syndromes (“*Xue*-stasis”, “phlegm”, “Liver yang hyperactivity”, and “endogenous liver wind”) and with the acupuncture reduction manipulation.

The needles were manipulated by way of the respective method after insertion, after 15 minutes and before extraction after 30 minutes. All acupuncture treatments were administered by the same physician acupuncturist in IA + SR group. Point location and depth of insertion were performed as described in standard textbooks [30]. Disposable sterile steel needles of 0.30 × 30 mm were inserted to a depth of 10–30 mm.

### 2.3. Outcome Measurements

#### 2.3.1. Primary Outcome Measurement

*Barthel Index (BI)*. The Barthel Index (BI), also known as the Barthel Scale or Barthel ADL Index, is a scoring tool of a person's performance in the activities of daily life (ADL). It is used, in particular, to measure the self-care ability of patients with neurological disabilities such as those following a stroke. Patients were assessed in 10 areas of daily activities, including feeding, moving from a wheelchair to a bed and back, grooming, transferring to and from a toilet, bathing, walking on a level surface, going up and down stairs, dressing, and is continence of bowels and bladder. A score of 0–10 given for each of the 10 areas, depending on the patient's performance level, with a higher score indicating better performance, yielding a total score of 0–100. Besides being a quick and easy tool to provide an objective assessment of a patient's ADL, the Barthel Index is robust in that it has demonstrated high interrater reliability (0.95) and test retest reliability (0.89) as well as high correlations (0.74–0.8) with other measures of physical disability [31]. 

#### 2.3.2. Secondary Outcome Measurements

*FM Motor and Sensory Assessment*. The Fugl-Meyer (FM) Motor and Sensory Assessments are used to measure the performance of voluntary limb movement (50 items) and the level of limb sensation (12 items), respectively. Each item is assessed and scored on a 3-point ordinal scale of 0–2 (0 for no performance/absent sensation, 1 point for partial performance/impaired sensation and 2 points for complete performance/normal sensation). 

The FM motor assessment (50 items, total FM motor score of 100), in turn, consists of the upper extremity (UE) subscale (33 items, score range of 0–66) and the lower extremity (LE) subscale (17 items, score range of 0–34). 

The FM sensory assessment (12 items, total FM sensory score of 24), in turn, consists of the light touch subscale (2 items each for UE and LE, score range of 0–8) and the proprioception subscale (4 items each for UE and LE, score range of 0–16) [32].

*European Organization for Research and Treatment of Cancer (EORTC) Core Quality of Life Questionnaire (QLQ-C30) and Brain Cancer Module (BCM20)*. The standard Chinese version of the EORTC QLQ-C30 has been used to assess the quality of life (QOL) of patients. It is a 30-item questionnaire consisting of multi-item subscales and single items that cover various dimensions of QOL. Subscales include 5 functional subscales (physical, role, cognitive, emotional, and social); 3 symptom subscales (fatigue, pain, and nausea/vomiting); a global health/QOL subscale. Single items include items for the assessment of typical symptoms reported by cancer patients (dyspnea, appetite loss, sleep disturbance, constipation, and diarrhea) and an item related to the perceived financial impact of cancer and cancer treatment. The “yes/no- responses” are employed in the physical functional subscales. The “modified 7-point linear analogue scales” is employed in the global health/QOL subscales. The “4-point Likert-type scales”, ranging from “1-not at all” to “4-very much”, is employed in all other items. This questionnaire has demonstrated adequate reliability in studies related to patients with brain cancer [33]. It takes, on the average, 8 minutes to complete. Moreover, the compliance rates in multicenter and phase III clinical trials are very high for both the full questionnaire and individual item completion.

The BCM, a supplement to the QLQ-C30, is a new questionnaire developed for brain cancer patients, which addresses symptoms that are specific to brain cancer or its treatment. The 20-item EORTC brain cancer-specific questionnaire (QLQ-BN20) is organized into four scales: future uncertainty (3 items), visual disorder (3 items), motor dysfunction (3 items), and communication deficits (3 items), as well as 7 single items related to headache, seizures, drowsiness, hair loss, itching, weakness of both legs; and difficulties with bladder control. The “four-point Likert-type scales” (“not at all”, “a little”, “quite a bit” and “very much”) is employed and is linearly transformed to a 0–100 scale, with higher scores indicating more severe symptoms [34].

FM and QLQ-C30-BN20 assessments were carried out before treatment and after an 8-week intervention. The Barthel scale was signed every week after intervention. An independent physician who performed the outcome assessments was blinded to treatment assignments.

### 2.4. Statistical Analysis

Statistical analysis was performed with SPSS version 17.0 (SPSS Inc., Chicago, IL, USA). The analysis was on a per-protocol basis. Descriptive statistics are presented as arithmetic means, standard deviation (SD), and 95% confidence intervals (95% CI) for the sake of clarity. *χ* ^2^ test, *t*-test, one-way, and repeated measures data of ANOVA test which were used to compare the demographic characteristics and other variables of the 3 groups where appropriate. A value of *P* < 0.05 was considered significant. Microsoft Office Excel 2010 (Microsoft Corporation, Redmond, WA, USA) was used for the graphical presentation of the results. This analysis was restricted to the participants who fulfilled the protocol, known as per-protocol analysis. 

We determined the sample size based on detecting the difference in the motor dysfunction of the BCM20 scale, with a power of 90% and a CI of 95%. The formula *n* = 2[(*Z*_*α*/2_+*Z*_*β*_)*σ*/*δ*]^2^ was used, with *α* = 0.05, *β* = 0.90, *δ* = 38.7 − 11.1 = 27.6, and *σ* = 23.1, which were chosen based on a previous study [35]. The estimated sample sizes were 20 (per group) in our study with a 15% dropout rate.

## 3. Results

### 3.1. Baseline Characteristics

For this pilot study a total of 58 patients were enrolled and randomized to either the IA + SR group (*n* = 19), the SA + SR group (*n* = 19), or the SR group (*n* = 20) in the present study. Patient baseline characteristics were comparable between the three groups (Table 3). There were no significant differences found between the three groups for age, gender, hemiplegia type, or extent of resection (all *P* > 0.05). None of the patients developed adverse effects caused by the acupuncture treatment.

Five patients dropped out during the trial, accounting for an 8.62% dropout rate. There were no deaths or protocol violators during the trial. Among the 5 dropouts, 2 patients discharged earlier because of economic problems; 2 patients defaulted on treatments; 1 patient withdrew and had a referral for radiotherapy or chemotherapy because the progressive tumor was growing. There was no statistical difference in between the dropouts from the 3 groups. 

### 3.2. Barthel Index Scores

A repeated measure analysis of variance (ANOVA) model was used to compare the time-course profiles of the three groups with regard to the Barthel index scores. 

The repeated measure analysis of variance between the groups showed significant differences (*F* = 7.771, *P* = 0.001). The LSD test showed significantly higher Barthel index scores in the IA + SR group than in the SR group from the 4th week to the 8th week after intervention (*P* < 0.05) and also higher scores, than in the SA + SR group from the 5th week to the 8th week (*P* < 0.05). It was also found that there was a statistically significant difference in the Barthel index score between the SA + SR group and the SR group at the 6th week (*P* = 0.049, 95% CI 0.04–19.56), while there were no significant differences between the two groups for BI scores at other weeks (*P* > 0.05 for all). By repeated measure analysis of variance, the Barthel index scores actually showed significant differences according to the sampling time (*F* = 46.096, *P* < 0.001). Similar results were, respectively, observed in the IA + SR group (*F* = 35.066, *P* < 0.001), the SA + SR group (*F* = 21.183, *P* < 0.001), and the SR group (*F* = 3.608, *P* = 0.005). The Barthel index score in each group was the lowest in the pretreatment phase and then gradually increased after the intervention and reached a peak at the 8th week.

**Figure 2:**
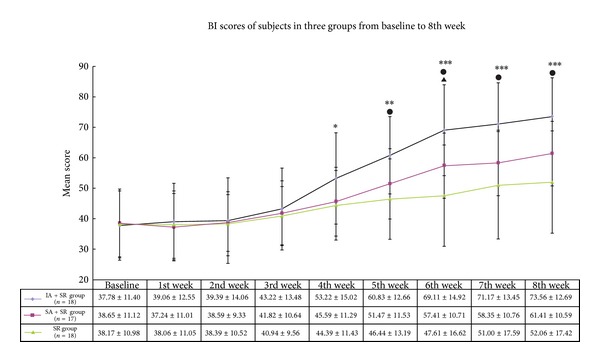
BI scores from the baseline to the 8th week. LSD post-hoc testing showed significant difference according to sampling time. “∗”: comparison between the IA + SR group and the SR group (*P < 0.05; **P < 0.01; ***P < 0.001); “●”: comparison between the IA + SR group and the SA + SR group (^●^ *P* < 0.05); “▲”: comparison between the SA + SR group and the SR group (^▲^ *P* < 0.05). The numbers are the means ± standard deviation.

 A group × time interaction was found between the group over time (ANOVA for Repeated Measure, Group-Time, *F* = 3.643, *P* = 0.001) (Figure 2).

### 3.3. Fugl-Meyer Assessment of Sensorimotor Function

By ANOVA we found that there were no significant differences at the baseline for the scores of FM (upper extremity), FM (lower extremity), FM (light touch), FM (proprioception), FM (total motor), and FM (total sensory) (*P* > 0.05, for all) between the three groups.

All groups showed significant changes on FM motor assessments (upper extremity and total motor) by comparison of scores at baseline and after 8 weeks (*t* test, *P* < 0.05, for all), while the score of FM motor assessment (lower extremity) significantly improved in the IA + SR group (*t* test, *P* < 0.05).

All scores of FM sensory assessments (light touch, proprioception, total sensory) significantly improved in the IA + SR group (*t* test, *P* < 0.05, for all), while in the SA + SR group, only the total sensory FM score significantly improved (*t* test, *P* < 0.05), while in the SR group, the scores of FM sensory assessments (light touch, proprioception, total sensory) did not significantly improve from the baseline to 8 weeks (*t* test, *P* > 0.05, for all) (Table 4).

**Figure 3:**
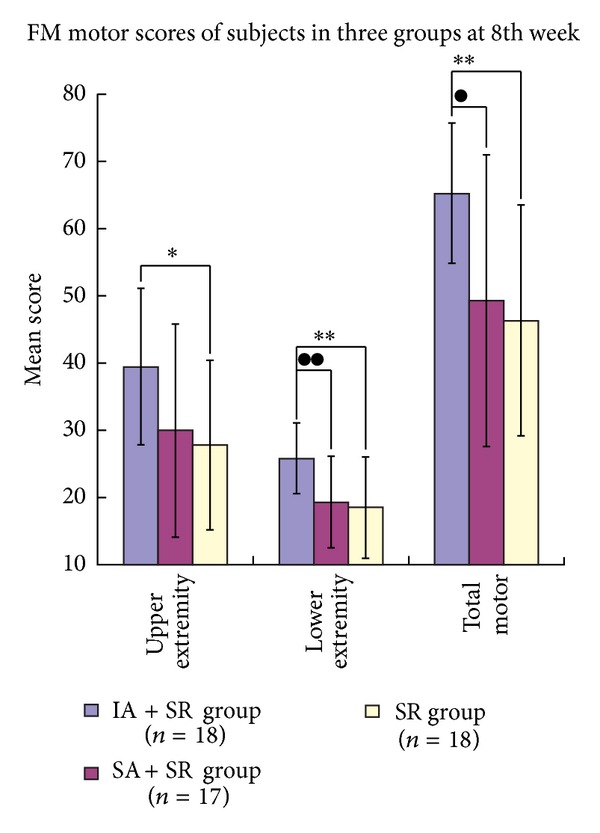
Comparison of FM motor scores of the 3 groups at 8th week. The post-hoc testing showed a significant difference: “∗”: comparison between the IA + SR group and the SR group (*P < 0.05; **P < 0.01); “●”: comparison between the IA + SR group and the SA + SR group (^●^ *P* < 0.05; ^●●^ *P* < 0.01).

Significant differences were observed at the 8th week for the scores of FM upper extremity (*F* = 3.822, *P* = 0.029), FM lower extremity (*F* = 6.533, *P* = 0.003), and FM total motor (*F* = 6.463, *P* = 0.003) between the three groups (Table 4).  Figure 3 shows the comparison of the posttreatment motor scores of the three groups: significant differences were found in FM (upper extremity) scores in comparison between the IA + SR group and the SR group (Tamhane test, *P* = 0.020, 95% CI 1.52–21.82); in FM (lower extremity) scores in comparison between the IA + SR group and the SA + SR group (LSD's test, *P* = 0.006, 95% CI 1.99–10.97) and between the IA + SR group and SR group (LSD's test, *P* = 0.002, 95% CI 2.86–11.70); FM (total motor) scores comparison between IA + SR group and SA + SR group (Tamhane test, *P* = 0.034, 95% CI 1.00–30.97) and between IA + SR group and SR group (Tamhane test, *P* = 0.001, 95% CI 6.94–30.95). No significant differences were observed between the SR group and the SA + SR group in FM (upper extremity), FM (lower extremity), and FM (total motor) (*P* > 0.05, for all).

**Figure 4:**
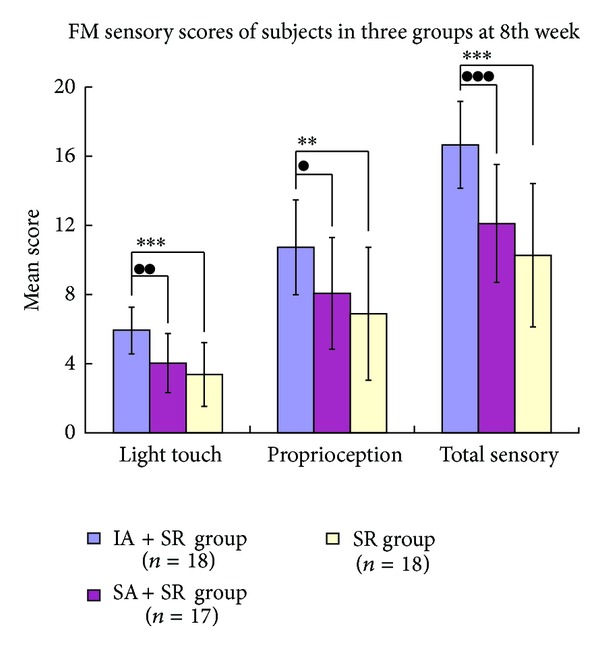
Comparison of FM sensory scores of the three groups at the 8th week. The post-hoc testing showed a significant difference: “∗”: comparison between the IA + SR group and the SR group (**P < 0.01; ***P < 0.001); “●”: comparison between the IA + SR group and the SA + SR group (^●^ *P* < 0.05; ^●●^ *P* < 0.01; ^●●●^ *P* < 0.001).

Significant differences between the three groups were observed at the 8th week for the scores of FM light touch (*F* = 11.575, *P* = 0.000), FM proprioception (*F* = 6.346, *P* = 0.003), and FM total sensory (*F* = 16.594, *P* = 0.000) (Table 4).  Figure 4 shows the comparison of posttreatment sensory scores of the three groups by the LSD test: FM (light touch) scores are comparable between the IA + SR group and the SA + SR group (*P* = 0.001, 95% CI 0.76–3.01) as well as between the IA + SR group and the SR group (*P* = 0.000, 95% CI 1.45–3.66); FM (proprioception) scores are comparable between the IA + SR group and the SA + SR group (*P* = 0.021, 95% CI 0.42–4.91) and also between the IA + SR group and the SR group (*P* = 0.001, 95% CI 1.62–6.05); FM (total sensory) scores are comparable between the IA + SR group and the SA + SR group (*P* = 0.000, 95% CI 2.22–6.87) as well as between the IA + SR group and the SR group (*P* = 0.000, 95% CI 4.10–8.68). Meanwhile, there were no significant differences between the SR group and the SA + SR group in FM light touch, FM proprioception, and FM total sensory (*P* > 0.05, for all).

### 3.4. EORTC QLQ-C30-BN20

With respect to the QLQ-C30-BN20 scores there were no significant differences between the three groups in overall scores at the baseline (ANOVA, *P* > 0.05, for all) (Table 5).

**Figure 5:**
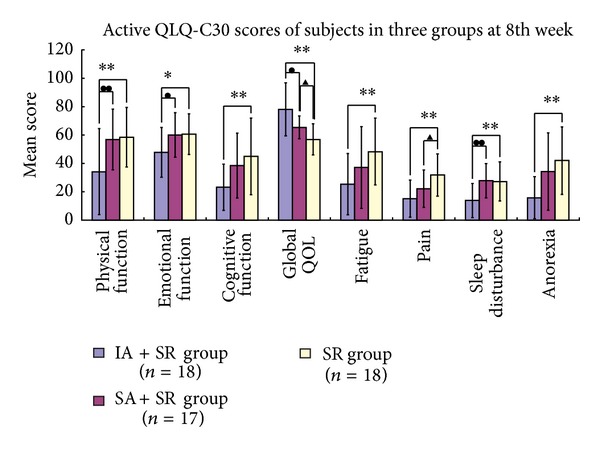
Active QLQ-C30 functions, symptoms, and global QOL scores at the baseline and at the 8th week. The post-hoc testing showed a significant difference: “∗”: comparison between the IA + SR group and the SR group (*P < 0.05; **P < 0.01); “●”: comparison between the IA + SR group and the SA + SR group (^●^ *P* < 0.05; ^●●^ *P* < 0.01); “▲”: comparison between the SA + SR group and the SR group (^▲^ *P* < 0.05).

Figure 5 summarizes significant differences between the three groups at 8 weeks after the intervention in the domains of physical function (ANOVA, *F* = 5.404, *P* = 0.008), emotional function (ANOVA, *F* = 3.720, *P* = 0.031), cognitive function (ANOVA, *F* = 4.431, *P* = 0.017), and global QOL (ANOVA, *F* = 11.347, *P* < 0.001) and in symptoms of fatigue (ANOVA, *F* = 3.815, *P* = 0.029), pain (ANOVA, *F* = 6.632, *P* = 0.003), insomnia (ANOVA, *F* = 6.995, *P* = 0.002), and anorexia (ANOVA, *F* = 6.485, *P* = 0.003). It also details significant differences of posttreatment through the posthoc tests: physical function between the IA + SR group and the SR group (LSD, *P* = 0.005, 95% CI 7.76–40.80) and between the SA + SR group and the SR group (LSD, *P* = 0.009, 95% CI 5.89–39.42); emotional function between IA + SR group and SR group (LSD, *P* = 0.019, 95% CI 2.20–23.58) as well as between the SA + SR group and the SR group (LSD, *P* = 0.027, 95% CI 1.49–23.18); cognitive function between the IA + SR group and the SR group (LSD, *P* = 0.006, 95% CI 6.69–36.76); global QOL between the IA + SR group and the SR group (Tamhane, *P* = 0.001, 95% CI 8.18–34.16) and between the IA + SR group and the SA + SR group (Tamhane, *P* = 0.043, 95% CI 0.35–25.06) as well as between the SA + SR group and the SR group (Tamhane, *P* = 0.040, 95% CI 0.31–16.62); fatigue between the IA + SR group and the SR group (LSD, *P* = 0.008, 95% CI 6.21–39.34); pain between the IA + SR group and the SR group (LSD, *P* = 0.001, 95% CI 7.44–25.89) and between the SA + SR group and the SR group (LSD, *P* = 0.045, 95% CI 0.24–18.97); insomnia between the IA + SR group and the SR group (LSD, *P* = 0.002, 95% CI 5.00–21.89) and between the IA + SR group and the SA + SR group (LSD, *P* = 0.002, 95% CI 5.36–22.50); anorexia between the IA + SR group and the SR group (Tamhane, *P* = 0.001, 95% CI 9.49–42.95). 

**Figure 6:**
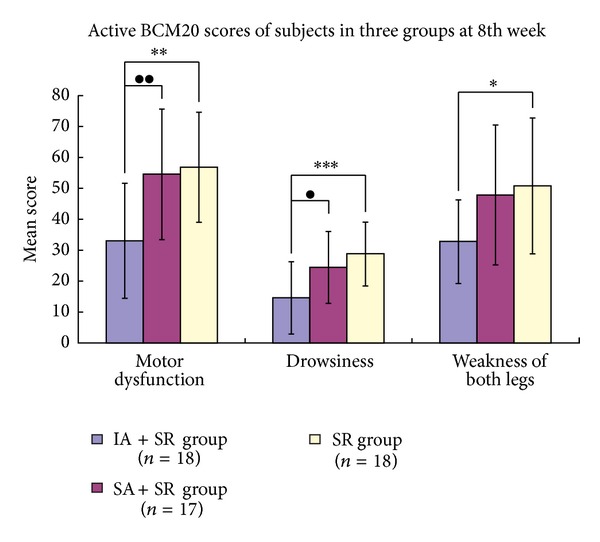
Active BCM20 scores at the baseline and the 8th week. The post-hoc testing showed a significant difference: “∗”: comparison between the IA + SR group and the SR group (*P < 0.05; **P < 0.01; ***P < 0.001); “●”: comparison between the IA + SR group and the SA + SR group (^●^ *P* < 0.05; ^●●^ *P* < 0.01).

Figure 6 shows that there were significant differences after intervention with respect to BN20 scores between the three groups in motor dysfunction (ANOVA, *F* = 8.365, *P* = 0.001), drowsiness (ANOVA, *F* = 7.506, *P* = 0.001), and weak legs (ANOVA, *F* = 4.285, *P* = 0.019). The post-hoc tests detected significant changes in motor dysfunction between the IA + SR group and the SR group (LSD, *P* = 0.001, 95% CI 10.93–36.63) and between the IA + SR group and the SA + SR group (LSD, *P* = 0.002, 95% CI 8.49–34.56); in drowsiness between the IA + SR group and the SR group (LSD, *P* < 0.001, 95% CI 6.65–21.69) and between the IA + SR group and the SA + SR group (LSD, *P* = 0.012, 95% CI 2.22–17.48); and in weak legs betweenthe IA + SR group and the SR group (Tamhane, *P* = 0.018, 95% CI 2.58–33.42).

The post-hoc tests show that there were no significant differences between the SA + SR group and the SR group for scores of items of QLQ-C30 and BCM20 scales (*P* > 0.05, for all) except with regard to the global quality of life and pain.

Compared with pretreatment in the IA + SR group tested by paired *t*-test, the scores significantly decreased after intervention in functional subscales of QLQ-C30 (physical, cognitive, and emotional) and symptom subscales (fatigue, pain, insomnia, and anorexia) (*P* < 0.05 for all) and in scales/items of BCM20 (future uncertainty, motor dysfunction, drowsiness, and weakness of both legs) (*P* < 0.05 for all). The score of a global QOL subscale, however, significantly improved after intervention in the IA + SR group and the SA + SR group (*P* < 0.05 for all). Significant changes were found in scores of cognitive function and symptom subscales (fatigue, pain, insomnia and anorexia) of QLQ-C30 and drowsiness of BCM20 for the SA + SR group (*P* < 0.05 for all). 

## 4. Discussion

Our pilot study shows the first evidence that acupuncture may play a role in the rehabilitation of hemiparesis in patients with astrocytomas. The results show that either standard and individualized acupuncture or routine physiotherapy treatment can significantly improve motor functions in upper and lower extremities, but individualized acupuncture was more effective on the lower extremity and global motor functions than standard acupuncture and standard rehabilitation without acupuncture, which was confirmed by the motor function in BCM20 scales. The results also support prior acupuncture studies on patients with hemiparesis after a stroke [10–12]. In Chinese medicine comparable concepts are used for treatment of hemiparesis after a stroke and due to brain tumors. This is not very surprising, because in western physiotherapy similar concepts for hemiparesis due to different causes are used. This might be an indication that certain parts of the mechanism of acupuncture in the rehabilitation of hemiparesis are, like western physiotherapy, the activation of healthy brain tissue around the lesions or activation of reciprocal inhibition [36] or perhaps even an enhancement of neuroplasticity [37, 38].

Placebo controlled studies in the rehabilitation of hemiparesis patients are almost impossible because all known therapies require an activation of the paralyzed limb. This includes acupuncture where, depending on the syndrome, reducing or reinforcing manipulation is necessary, while a neutral needling might not be stimulus enough [39]. A concept with placebo approaches like the Streitberger needle [40] seems inadequate for hemiparesis with differences of muscle tones. Randomization of patients, blinding the patients to the concept of acupuncture and blinding the independent observers to the therapeutic procedure as done in this study, seems adequate for studies on hemiparesis with acupuncture. 

A general problem with acupuncture studies is the standardization of the procedures, while in traditional Chinese medicine an individualized approach with personalized acupuncture treatment is expected. We solved this problem by using two groups, one with a standardized concept that was promoted for studies on hemiparesis before [24–26] and the other with a unique, individualized concept taking the prominent area of symptoms and the status of the muscle tones into account. Our data showed the superiority of the individualized concept, which supports the necessity of individualized treatments and contradicts studies where there were not any different treatment effects found, regardless of where the needles were inserted [41]. One disadvantage of different treatment options in an individualized treatment protocol is that it is hard to distinguish which acupoint has the specific effect. To study this, much bigger studies with the possibility of subgroup analysis are necessary. Prior studies of our group showed evidence that electroacupuncture at *Ren* and *Du* meridians can inhibit the overproliferation of hippocampal horizontal astrocytes after focal cerebral ischemia and promote the astrocytes differentiation in an animal model [42, 43]. Needling techniques on *Ren* and *Du* meridians had also been shown to improve brain stem auditory conducting function in patients with cerebral infarction by brain stem auditory evoked potential [44]. While activating of the *Ren* and *Du* meridians was part of the protocol of the individualized acupuncture group in this trial, similar mechanism of activation as well as inhibition of overproliferation might play a role especially in the treatment of spastic paresis, where we have a disbalance of activation and inhibition.

Our data shows that individualized acupuncture can improve the Barthel ADL index more than standard acupuncture and no acupuncture. Interestingly, significant differences between the tested groups just started after the treatment period of 4 weeks and became really significant after 8 weeks. This is similar to other clinical studies on the nervous system, where measurable improvements were seen in a period of more than 3 months [45]. This is not surprising, since activation of not completely damaged neural cells as well as activation of formerly nonactive cell takes time. This is important for future study designs: treatment as well as observation periods should be prolonged.

While in the nonacupuncture group no significant improvement of sensory function had been found, standard acupuncture improves only the global sensory function, and individualized acupuncture had a positive impact on global sensory impact, light touch, and proprioception. This is of importance since there is no other established treatment known having an effect on improving sensory function due to brain tumors beyond the natural improvement without specific treatment after intervention. 

QLQ-C30 scales showed improvement after treatment with individualized acupuncture in functions such as physical, emotional, cognitive, and global QL, and in symptoms of fatigue, insomnia, and anorexia. 

Our study also gave evidence that acupuncture can relieve pain in astrocytoma patients, but no significant difference between the standard and the individualized acupuncture was observed. 

Future studies with bigger groups with the possibility of subgroup analysis and longer treatment and observation periods for astrocytomas grade 2 are necessary. Then the transferral of these data to other brain tumor types can be tested. While previous studies showed evidence of a positive impact of Chinese herbs on the quality of life of brain tumor patients [46, 47], it might be possible to develop a complex complementary concept for brain tumor patients.

## 5. Conclusion

This study gives first evidence that acupuncture might play a relevant role in the rehabilitation of astrocytoma grade II patients with hemiparesis. Individualized acupuncture plus standard rehabilitation was superior to standardized acupuncture plus standard rehabilitation and to standard rehabilitation alone. We conclude that the individualized acupuncture concept of “*connecting and regulating Ren and Du*” combined with “*Jin-3-needling*” offers a promising possibility for the treatment of hemiparesis due to astrocytoma, but further evaluation is mandatory.

## Figures and Tables

**Table 1:** The protocol of *Jin-3-needling* for hemiparesis.

Not relevantly increased muscle tone	Upper limb	*Quchi (LI 11), Hegu (LI 4), Waiguan (SJ 5) *
	Lower limb	*Zusanli (ST 36), Sanyinjiao (SP 6), Taixi (KI 3) *
Spasticity	Upper limb	*Neiguan (PC 6), Jiquan (HT 1), Chize (LU 5) *
	Lower limb	*Chongmen (SP 12), Weizhong (BL 40), Zhaohai (KI 6) *
Spasticity or not relevant increased muscle tone	Shoulder	*Jianyu (LI 15) *and 2 extrapoints [2* cun* posterior and anterior of *Jianyu (LI 15)*]

**Table 2:** Additional acupuncture based on syndromes.

Syndrome [29]	Selected points	Operation
*Qi* deficiency	*Guanyuan (RN 4)*, *Qihai (RN 6) *	Additional moxibustion with the reinforcing method
*Xue* stasis	*Xuehai (SP 10) *	Bilateral acupuncture with the reducing method
Phlegm	*Fenglong (ST 40) *	Bilateral acupuncture with the reducing method
*Yin* deficiency	*Taixi (KI 3) *	Bilateral acupuncture with the reinforcing method
Liver yang hyperactivity	*Fengchi (GB 20) *	Bilateral acupuncture with the reducing method

**Table 3:** Characteristics of patients that completed the study in 3 groups.

Groups	IA + SR	SA + SR	SR
Characteristics	*N* = 18	*N* = 17	*N* = 18
Mean age (range), yr	44.21 ± 10.66 (25~65)	43.74 ± 11.26 (23~64)	43.99 ± 10.57 (20~62)
Gender (women/men)	6/12	7/10	7/10
Side of hemiplegia (right/left)	8/10	6/11	7/11
Extent of resection			
<50%	5	6	5
50–89% resection alone	4	4	5
Macroscopic removal (>90%)	9	7	8

**Table 4:** Fugl-Meyer assessment of sensorimotor function at baseline and at 8th week.

	IA + SR group (*n* = 18)		SA + SR group (*n* = 17)		SR group (*n* = 18)		*P* value (ANOVA)	
	Pretreatment	8th week later	Pretreatment	8th week later	Pretreatment	8th week later	Pretreatment	8th week later
FM motor assessment	35.56 ± 15.48	65.28 ± 10.45*	29.82 ± 16.21	49.29 ± 21.72*	29.94 ± 13.31	46.33 ± 17.12*		0.003^#^
Upper extremity subscore (0–66)	17.67 ± 12.01	39.44 ± 11.64*	16.47 ± 10.28	29.94 ± 15.88*	16.44 ± 8.72	27.78 ± 12.59*		0.029^#^
Lower extremity subscore (0–34)	13.89 ± 6.90	25.83 ± 5.28*	13.35 ± 6.38	19.35 ± 6.80	13.50 ± 5.83	18.56 ± 7.54	*P* > 0.05 for all	0.003^#^
FM sensory assessment	9.94 ± 2.73	16.67 ± 2.52*	9.53 ± 1.77	12.12 ± 3.41*	9.56 ± 2.01	10.28 ± 4.14		0.000^#^
Light touch subscore (0–8)	3.22 ± 2.16	5.94 ± 1.35*	3.18 ± 2.24	4.06 ± 1.71	3.28 ± 1.90	3.39 ± 1.85		0.000^#^
Proprioception subscore (0–16)	6.72 ± 2.93	10.72 ± 2.74*	6.35 ± 3.69	8.06 ± 3.23	6.28 ± 3.59	6.89 ± 3.85		0.003^#^

*P < 0.05, compared with pretreatment in each group by paired *t*-test, ^#^ *P* < 0.05, comparison between the three groups by ANOVA. The numbers are the means ± standard deviation.

**Table 5:** EORTC QLQ-C30-BN20 scores in 3 groups at baseline and 8th week.

Questionnaire	Scales/Items	IA + SR group (*n* = 18)		SA + SR group (*n* = 17)		SR group (*n* = 18)		*P* value (ANOVA)	
		Baseline	8th week	Baseline	8th week	Baseline	8th week	Baseline	8th week
QLQ-C30	*Function scales *								
	Physical function	70.78 ± 19.07	34.11 ± 30.36*	64.65 ± 25.64	56.76 ± 21.35	60.83 ± 28.45	58.39 ± 20.98		0.008^△^
	Role function	41.72 ± 17.76	40.50 ± 17.56	46.12 ± 14.72	44.35 ± 16.44	44.83 ± 15.25	43.94 ± 15.86		0.751
	Emotional function	65.22 ± 15.85	47.72 ± 17.57*	64.12 ± 17.48	60.06 ± 15.76	63.78 ± 17.02	60.61 ± 14.39		0.031^△^
	Cognitive function	47.78 ± 13.49	23.17 ± 16.26*	47.47 ± 18.36	38.41 ± 22.86*	49.39 ± 17.36	44.89 ± 26.96		0.017^△^
	Social function	60.00 ± 23.21	59.44 ± 23.48	53.47 ± 20.09	53.00 ± 20.62	55.56 ± 16.72	53.50 ± 20.64		0.617
	Global QL	56.78 ± 17.46	78.06 ± 18.68*	53.35 ± 15.34	65.35 ± 7.93*	50.28 ± 8.16	56.89 ± 11.02*		0.000^△^
	*Symptom scales *							*P* > 0.05 for all	
	Fatigue	46.94 ± 15.70	25.44 ± 21.56*	50.12 ± 20.11	37.06 ± 28.91*	51.33 ± 19.16	48.22 ± 23.42		0.029^△^
	Nausea/vomiting	22.33 ± 7.67	21.56 ± 8.91	21.71 ± 8.01	20.59 ± 9.13	21.89 ± 7.35	22.06 ± 11.00		0.903
	Pain	38.83 ± 8.07	15.06 ± 13.14*	37.94 ± 7.52	22.12 ± 13.17*	35.89 ± 9.54	31.72 ± 14.92		0.003^△^
	Dyspnea	26.89 ± 16.22	26.06 ± 16.67	34.35 ± 8.25	33.59 ± 9.82	31.72 ± 9.69	30.56 ± 11.73		0.241
	Insomnia	31.06 ± 7.76	13.83 ± 11.97*	32.06 ± 6.89	27.76 ± 12.03*	29.94 ± 7.55	27.28 ± 13.74		0.002^△^
	Anorexia	45.72 ± 12.89	15.72 ± 14.83*	46.71 ± 20.39	34.24 ± 27.20*	45.94 ± 19.86	41.94 ± 23.73		0.003^△^
	Constipation	23.56 ± 8.18	23.11 ± 8.70	25.35 ± 14.06	25.00 ± 14.35	24.39 ± 14.61	24.28 ± 16.53		0.917
	Diarrhea	20.22 ± 7.16	19.78 ± 7.79	21.53 ± 19.48	20.94 ± 19.87	22.06 ± 15.27	21.94 ± 19.80		0.927
	Financial impact	78.11 ± 10.35	64.72 ± 29.70	77.94 ± 10.78	76.65 ± 12.45	77.61 ± 12.25	78.06 ± 10.68		0.095

BN-20	*Scales *								
	Future uncertainty	51.06 ± 32.45	32.22 ± 19.77*	43.88 ± 31.98	28.94 ± 22.24	45.56 ± 30.26	30.39 ± 21.28		0.899
	Visual disorder	18.83 ± 20.71	17.17 ± 20.81	21.12 ± 21.66	18.76 ± 20.10	21.17 ± 19.71	19.28 ± 19.33		0.948
	Motor dysfunction	68.56 ± 16.48	32.94 ± 18.61*	64.12 ± 17.48	54.47 ± 21.14	65.67 ± 16.83	56.72 ± 17.79		0.001^△^
	Communication deficit	21.17 ± 23.51	15.06 ± 16.34	17.06 ± 16.98	16.35 ± 16.29	22.06 ± 21.71	21.11 ± 17.94		0.532
	*Items *							*P* > 0.05 for all	
	Headaches	17.28 ± 24.76	13.06 ± 19.88	14.35 ± 21.75	14.06 ± 20.24	14.61 ± 20.76	14.50 ± 19.62		0.975
	Seizures	12.06 ± 15.19	11.17 ± 12.90	10.71 ± 15.30	11.65 ± 15.15	12.56 ± 17.20	11.94 ± 14.75		0.987
	Drowsiness	33.22 ± 13.99	14.44 ± 11.75*	37.71 ± 19.94	24.29 ± 11.55*	36.50 ± 20.01	28.61 ± 10.37		0.001^△^
	Bothered by hair loss	9.39 ± 7.67	9.33 ± 7.59	9.29 ± 7.90	10.24 ± 7.77	8.89 ± 7.81	8.83 ± 7.34		0.858
	Bothered by itchy skin	10.44 ± 11.76	10.39 ± 11.54	9.94 ± 12.22	10.29 ± 12.11	10.28 ± 11.90	11.17 ± 12.42		0.972
	Weakness of both legs	64.78 ± 15.58	32.67 ± 13.46*	56.12 ± 24.07	47.71 ± 22.61	60.17 ± 19.19	50.67 ± 21.97		0.019^△^
	Trouble controlling bladder	18.11 ± 24.75	11.67 ± 19.01	12.06 ± 18.20	13.82 ± 20.41	18.39 ± 20.04	17.72 ± 19.37		0.646

*P < 0.05, compared with pretreatment in each group by paired *t*-test, ^△^ *P* < 0.05, comparison between the three groups by ANOVA. The numbers are the means ± standard deviation.

## Abbreviations

ADL: Activities of daily life
ANOVA: A repeated measures analysis of variance
BCM20: 20-item brain cancer module
BI: Barthel index
BL: *Taiyang* bladder meridian of the foot
CNS: Central nervous system
DAS: Drug and Statistics
DU: *Du* meridian
EORTC: European Organization for Research and Treatment of Cancer
FM: Fugl-Meyer Motor and Sensory Assessment
HT: *Shaoyin* heart meridian of the hand
IA: individualized acupuncture
KPS: Karnofsky performance status
LU: *Taiyin* lung meridian of the hand
PC: *Jueyin* pericardium meridian of the hand
QLQ-C30: Core Quality of Life Questionnaire
QOL: Quality of life
RN: *Ren* meridian
SA: Standard acupuncture
SP: *Taiyin* spleen meridian of the foot
SR: Standard rehabilitation
ST: *Yangming* stomach meridian of the foot.
